# The Absence of Items Addressing Increased Appetite or Weight in Depressive-Symptom Questionnaires: Implications for Understanding the Link between Major Depressive Disorder, Antidepressants, and Obesity

**DOI:** 10.3390/brainsci14080841

**Published:** 2024-08-21

**Authors:** Andrés M. Treviño-Alvarez, Marci E. Gluck, Susan L. McElroy, Alfredo B. Cuellar-Barboza

**Affiliations:** 1 Department of Psychiatry, University Hospital and School of Medicine, Universidad Autonoma de Nuevo Leon, Monterrey 64460, NL, Mexico; trevinoalvarez.andres@mayo.edu; 2 Department of Psychiatry and Psychology, Mayo Clinic Arizona, Phoenix, AZ 85054, USA; 3 Department of Health and Human Services, Obesity and Diabetes Clinical Research Section, Phoenix Epidemiology and Clinical Research Branch, National Institute of Diabetes and Digestive and Kidney Diseases, National Institutes of Health, Phoenix, AZ 85016, USA; gmarci@niddk.nih.gov; 4 Lindner Center of Hope, Mason, OH 45040, USA; susan.mcelroy@lindnercenter.org; 5 Department of Psychiatry and Behavioral Neuroscience, University of Cincinnati College of Medicine, Cincinnati, OH 45267, USA; 6 Department of Psychiatry and Psychology, Mayo Clinic, Rochester, MN 55905, USA

**Keywords:** depressive symptoms, questionnaires, hunger, appetite, weight gain, antidepressants

## Abstract

Major depressive disorder (MDD) and obesity have a complex bidirectional relationship. However, most studies do not assess increased appetite or weight as a depressive symptom due to limitations in rating scales. Here we aimed to analyze frequently employed depressive-symptom scales and discuss the relevance of weight and appetite assessment items. To elaborate this perspective, we searched for validated questionnaires and scales evaluating depressive symptoms in English. We analyzed appetite and weight items from 20 depressive-symptoms rating scales. Only 8 of 20 rating scales assessed for increased weight or appetite. The scales reported in the literature as the most employed in antidepressants efficacy trials do not assess increased appetite or weight. The current use of rating scales limits our understanding of the relationship between MDD, antidepressants, and obesity. It is necessary to improve our weight and appetite measurements in MDD to clarify the respective impact of depressive symptoms and antidepressants on weight change.

## 1. Introduction

Major depressive disorder (MDD) is a common mental health disorder characterized by a depressive mood and anhedonia, and loss of functionality, and may present thoughts of death [1]. Obesity is defined as a chronic state of excessive accumulation of adipose tissue, with a disproportion of body weight to height, accompanied by an inflammatory state [2]. It is now well recognized that MDD and obesity are related. Meta-analytic evidence has shown an association between diagnosed MDD and obesity incidence [3] and a bidirectional relationship between depressive symptoms and obesity (depressive symptoms can lead to obesity and vice versa) [4]. MDD was reported as a risk factor with “convincing evidence” associated with obesity in adults (OR 1.58, 95%CI 1.33–1.87) in an umbrella review of meta-analyses [5]. Moreover, MDD has been associated with altered eating behavior [6] as well as a higher risk for type 2 diabetes (OR 1.49, 95%CI 1.29–1.72) [7] and metabolic syndrome (OR 1.54, 95%CI 1.21–1.97) [8]. These two conditions are strongly related to obesity.

Regarding depressive characteristics, anhedonia traits in healthy individuals were recently associated with a metabolic profile less prone to weight gain. Still, as self-reported depressive symptoms increased, this correlation decreased and was no longer significant [9]. However, the mechanisms underlying the MDD–obesity relationship are unclear and require integrating the complexity of eating behavior and metabolism [10]. Here, we comment on the relevance and limitations of eating behavior assessments in MDD studies and how depressive-symptoms questionnaires may influence the gap in knowledge regarding the relationship between MDD and obesity. We searched in PubMed for validated questionnaires and scales evaluating depressive symptoms in English. Our aim was to include those widely employed, including self- and clinicians’ assessments.

## 2. Eating Behavior in Major Depressive Disorder

MDD episodes often present with changes in appetite, eating behavior, and body weight, including increased appetite and weight gain (sometimes referred to as atypical symptoms or features). Most diagnostic criteria for an MDD episode include increased appetite or increased weight (DSM-5 [11], ICD-11 [12]) as defining criteria, and atypical symptoms are associated with weight gain and obesity [13]. On the other hand, MDD has been reported as the most frequent comorbidity in binge eating disorder [14] and bulimia nervosa, as well as anorexia nervosa [15]. A study reported that patients with MDD (remitted and with a current episode) had higher emotional and external eating than controls. Also, these eating behavior symptoms increased as depressive symptoms became more severe [16].

Both biological and behavioral pathways have been broadly described as possible mechanisms leading to obesity in MDD [17]. The former may include alterations in diverse systems such as the hypothalamic–pituitary–adrenal (HPA) axis, inflammatory system, neuropeptides, neurotransmitters, and the gut–brain axis. Regarding HPA axis alterations, blunted responses of ACTH, lower density of CRH receptors, and higher CRH levels have been identified in persons with MDD [18]. The inflammatory cytokine and HPA axis activator, IL-6, is also elevated in MDD as well as in obesity [18]. Insulin and leptin, neuroendocrine regulators with major roles in energy metabolism, have also been found altered in MDD [19]. The behavioral pathway that may lead to obesity in MDD includes changes in physical activity, food and beverage consumption, substance abuse, and medications [17]. A bidirectional relationship between physical activity and MDD has been found in a Mendelian randomization study—MDD led to less physical activity and greater physical activity was a protective factor for MDD [20]. Food consumption has been studied in response to various influences, including food attentional bias, emotional eating, external eating, and restrained eating. These factors are not isolated but interplay in a complex process [10]. Indeed, functional connectivity (FC) studies have identified that changes in appetite are related to greater severity of depressive symptoms but that decreased and increased appetite results from different changes in FC [21]. This case–control study used fMRI to study changes in appetite during a depressive episode from the Marburg–Münster FOR 2107 Affective Disorder Cohort Study (MACS). The nucleus accumbens was considered the seed of the reward circuit to map associations with opposing changes in appetite. Reduced FC between the nucleus accumbens and insular ingestive cortex was related to increased appetite. In contrast, decreased appetite was related to reduced FC between the nucleus accumbens, the ventromedial prefrontal cortex, and the hippocampus [21]. Other FC studies have also found decreased connectivity in the insular region regarding eating behavior, such as decreased global connectivity in persons with obesity when drinking a milkshake [22].

Environmental factors have also been identified as risk factors for MDD [23] and obesity [24]. Extensive epidemiological data from the United States National Health and Nutrition Examination Survey (68.3% of those surveyed were overweight or obese) showed that persons with higher consumption of ultra-processed food were more likely to report depressive and anxious symptoms [25]. Even more, meta-analytic evidence from prospective studies demonstrated an increased risk of subsequent depression in persons with high consumption of ultra-processed food. Food insecurity has also been identified as a risk factor for depression [26], maladaptive eating behavior, and overeating [27], as well as decreased dietary adherence [28], demonstrating that both the quality of the food as well as availability and accessibility matter for mental health. Considering that eating behavior has been reported to play an important role in mental health, particularly in the context of depressive symptoms, this should be thoroughly assessed in clinical and research practices.

## 3. Unhealthy Lifestyle in Major Depressive Disorder

To study weight change, it is important to consider both energy intake and energy expenditure. Energy expenditure research in MDD is out of the scope of this viewpoint, but we will briefly discuss a focus on how MDD can affect energy expenditure. MDD has been associated with low levels of physical activity and high levels of sedentary behavior [29,30]. Research on subtypes of MDD and physical activity measured by an accelerometer was performed in a cohort study designed to investigate cardiovascular diseases and mental disorders from a Swiss community. They found that persons with the remitted combined atypical-melancholic subtype had a higher likelihood of being less physically active [31]. Indeed, the presence of anhedonia in MDD has been associated with worse quality of life and functionality with assessments that include physical activity [32]. Low physical activity and high sedentary behavior may reflect an imbalance of low energy expenditure with greater food intake, leading to weight gain [33].

Treatment strategies for MDD have increasingly considered the prescription of physical activity [34]. Participants prescribed with high-dose exercise in the Treatment with Exercise Augmentation for Depression (TREAD) study, a 12-week randomized clinical trial with prescribed exercise for persons with partial or no response to an SSRI, improved in motivation and anhedonia scores [35]. Meta-analytic evidence has demonstrated that exercise, particularly aerobic in moderate intensity, is effective in the treatment of MDD [36]. Even more, a sample Mendelian randomization study found a protective effect of supervised physical activity on MDD, but not self-reported exercise [37]. Indeed, the mechanisms associated to MDD such as inflammatory pathways, altered neuroplasticity, and structural alterations in particular brain regions (e.g., hippocampus) [1] have been reported to improve in different exercise trials [38]. Despite the growing recommendations of physical activity for persons with MDD, it is still to be known if these patients receive these instructions in medical consultations. Persons may face depression- and obesity-related stigma by primary care physicians that may negatively affect the quality of attention provided [39,40].

## 4. Antidepressants and Weight Change

As glutamatergic dysfunction has gained attention as a target to treat MDD, it is interesting that in a large European study, obesity was causally associated with downregulated glutamine—the most abundant amino acid in blood and primary precursors for glutamate [41]—and higher BMI and lower glutamine levels were causally linked to MDD [42]. Indeed, the glutamate–glutamine cycle between neurons and astrocytes plays a major role in regulating synaptic glutamate levels and excitatory transmission [41], and dysfunction in glutamate receptors during development may lead to neuropsychiatric diseases [43]. Studies have found decreased glutamine and increased glutamate levels in persons with obesity [42,44,45]. Accordingly, weight-loss interventions have been found to correct glutamine and glutamate levels [46]. Of interest, glucagon-like peptide-1 receptor agonists—known for beneficial effects on metabolism and weight control—may also improve depressive symptoms in individuals with diabetes or obesity [47,48], and glucagon-like peptide-1 receptors are stimulated by glutamine [49]. In contrast, many commonly used antidepressants are associated with weight gain [50].

A meta-analysis of 116 studies reported that paroxetine, mirtazapine, and amitriptyline had a greater risk for weight gain. Fluoxetine and bupropion were associated with weight loss, while other antidepressants had no significant effect on body weight [51]. Studies were classified into acute (4–8 weeks) and maintenance treatment (>4 months). Weight gain in maintenance treatment was significant for paroxetine (399 cases; 387 controls; mean difference, kg: 95%CI: 2.73 (0.78 to 4.68), *p* = 0.006) and amitriptyline (cases 170; controls 140; mean difference, kg: 95%CI: 2.24 (1.82 to 2.66) *p* < 0.001), and clinically relevant for mirtazapine (cases 559, controls 542, mean difference, kg, 95%CI: 2.59 (−0.23 to 5.41), *p* = 0.07). However, this association is confounded by several variables not included in this meta-analysis, such as appetite assessment at baseline or how many patients with weight gain either had or developed obesity. Similarly, longitudinal evidence links antidepressant prescription and weight gain. In a 10-year follow-up study with a population-based cohort, Gafoor et al. evaluated antidepressant utilization and weight gain using primary care electronic health records databases. In this extensive cohort (*n*= 136,762 men and 157,957 women), in 1,836,452 person-years of follow-up, an increased risk of weight gain (weight increase of ≥5% compared with the previous year) was found in antidepressant users (AOR: 1.21, 95%CI: 1.19–1.22, *p* < 0.001). The adjusted rate ratios of antidepressant prescription and changing from normal BMI to overweight or obesity was 1.29 (95% CI: 1.25–1.34), and from overweight to obesity was 1.29 (95%CI: 1.25–1.33). However, the authors clarify that they could not exclude the role of increased appetite or weight gain as primary depressive symptoms, which were not assessed, and did not report whether weight gain was healthy or pathological [52].

Few randomized controlled trials of antidepressants in MDD have explored appetite change and weight gain over the long term. An important exception is a one-year prospective study examining the long-term weight effects of fluoxetine in individuals with MDD remitting to 12 weeks of fluoxetine (20 mg/d). After remitting, patients were randomized for up to 38 weeks to placebo (*n* = 96) or fluoxetine continuation (14 weeks completed, *n* = 167; 26 weeks, *n* = 82; 38 weeks, *n* = 63). Modest weight loss was observed with fluoxetine during the 12 weeks of acute treatment (mean absolute weight decrease of 0.35 kg, *p* < 0.01) [53]. However, there was no significant difference in weight gain between placebo and fluoxetine groups in those who completed 50 weeks of treatment.

Additionally, appetite improvement after recovery from depression was associated with weight gain [53]. This study suggests that after recovery from depression, weight gain may occur, increasing over time, but is not likely to be caused by fluoxetine. The distinction between the intricate effect of both an increase in appetite from MDD and the antidepressant effect on weight gain requires more longitudinal studies designed to answer this question. Importantly, appetite and weight increase should be addressed before treatment initiation and followed throughout the placebo and intervention groups to determine if such complaints are due to MDD itself or a side effect of medication.

Regarding differences in treatment response, Quitkin and colleagues reported that patients with atypical depression symptoms (interpersonal sensitivity, lethargy, oversleeping, and overeating) responded better to phenelzine (MAOI) than imipramine (TCA)—even those with only a few atypical symptoms [54]. They argued that selective responsivity and symptomatology in these patients suggested a particular subgroup of MDD [54]. Kloiber et al. highlighted how patients with MDD and a greater BMI respond less well to antidepressants than patients with MDD and a normal BMI [55]. A retrospective analysis from an ongoing multi-center clinical study conducted by the European Group for the Study of Resistant Depression (GSRD) found that an elevated BMI was associated with higher suicidality, longer psychiatric hospitalizations, and earlier age of MDD onset, also reporting a statistical trend of higher BMI with treatment resistance [56]. Their limitations included a lack of more accurate markers of obesity, as the authors state. Still, we also consider as a limitation that their depressive-symptoms scale did not assess increased weight or appetite.

On the other hand, a recent meta-analysis found that the remission rate with antidepressants was higher in normal-weight to overweight patients than in patients with obesity. Analyzing subgroups, they found this was also true for monotherapy studies, but the remission rate in combined treatments (pharmacological) was higher in persons with obesity. However, their meta-regression analyses showed only a significant relationship between baseline BMI and remission rate in monotherapy (Q = 4.79, *p* = 0.029) but not in combined therapies (Q = 0.007, *p* = 0.98). These authors also made a call to consistently assess BMI in antidepressant studies since a large proportion of antidepressant studies lack BMI measurement and thus are excluded from these analyses [57]. They reported that the Hamilton Depression Rating Scale (HAM-D) and the Montgomery–Asberg Depression Rating Scale (MADRS) were the most frequent scales employed, both of which have an important limitation in weight assessment as described below.

## 5. Eating Behavior Assessment in Depressive-Symptoms Questionnaires

Many depression rating scales do not assess increased appetite or weight gain. Table 1 compares the appetite and weight items from validated clinician-administered and patient-reported depressive symptom scales. Zimmerman reported the HAM-D and the MADRS as the most frequently employed scales for symptom severity and efficacy in antidepressant efficacy trials [58]. However, the HAM-D [59] and the Beck Depression Inventory (BDI) [60] assess decreased appetite and weight, while the MADRS [61] only assesses decreased appetite—neither scale considers increased appetite or weight. Similarly, the Patient Health Questionnaire (PHQ-9) [62] and the Zung Self-Rating Depressive Scale [63] only present non-specific items referring to a “change in appetite.” Contrastingly, the Inventory for Depressive Symptomatology, Clinician Rated (IDS-C) [64], the Quick Inventory for Depressive Symptomatology, Self-Rated (QIDS-SR) [65], and the Symptoms of Depression Questionnaire (SDQ) [66] do include increased and decreased appetite and weight items. However, these scales are rarely used as primary outcome measures in pivotal trials of antidepressants in MDD. In 1996 a revision of the BDI was published (BDI-II) and here the appetite item was corrected to also ask about an increase in appetite [67].

Depressive-symptoms questionnaires have also been developed for an array of specific contexts; among these are the Remission from Depression Questionnaire (RDQ) [68], Edinburgh Postnatal Depression Scale (EPDS) [69], Geriatric Depression Scale (GDS) [70], Calgary Depression Scale for Schizophrenia (CDSS) [71], and Meno-D for menopause [72]. While metabolic alterations are found in schizophrenia onset even before medication use [73], and excessive weight gain in the third trimester of pregnancy is a risk factor for a depressive episode [74], only the RDQ and the Meno-D include items assessing appetite or weight gain. While eating behavior may not be the only explanation, assessment of these behavioral changes is vital better to describe the bidirectional relationship between weight gain and depression.

We posit that depression scales assessing for increased appetite and weight gain as well as reduced appetite and weight loss are essential for elucidating the MDD–obesity–antidepressant link. There is a significant knowledge gap regarding the use of antidepressants in the treatment of depressed individuals with atypical features or obesity due in part to the use of depression scales that only assess reduced appetite or decreased body weight as core depressive symptoms in pivotal trials. Indeed, we argue that the term atypical symptoms is misleading because increased appetite and weight gain are just as common as reduced appetite and weight loss in MDD [75].

**Table 1:** Comparison of appetite and weight items in validated depressive-symptoms scales.

INSTRUMENT	DECREASED		INCREASED	
	Appetite	Weight	Appetite	Weight
Inventory For Depressive Symptomatology, Clinician-Rated (IDS-C) [64]	Appetite (decreased)	Weight (decrease) within the last 2 weeks	Appetite (increased)	Weight (increased) within the last 2 weeks
Quick Inventory for Depressive Symptomatology. Self-Rated (QIDS-SR) [65]	Decreased appetite	Decreased weight (within the last two weeks)	Increased appetite	Increased weight (within the last two weeks)
Symptoms of Depression Questionnaire (SDQ) [66]	How has your appetite been over the past month?	Have you lost weight over the past month?	Has your appetite been excessive over the past month?	Have you gained weight over the past month?
Clinically Useful Depression Outcome Scale (CUDOS) [76]	My appetite was poor and I didn’t feel like eating.		My appetite was much greater than usual.	
Inventory of Depressive Symptoms [64]	Decreased appetite		Increased appetite	
The Remission from Depression Questionnaire (RDQ) [68]	My appetite was poor.		My appetite was much greater than usual.	
Beck Depression Inventory-II [67]	My appetite is somewhat less than usual. My appetite is much less than usual. I have no appetite at all.		My appetite is somewhat greater than usual. My appetite is much greater than usual. I crave food all the time.	
Beck Depression Inventory [60]	My appetite is no worse than usual	I haven’t lost much weight, if any, lately		
Hamilton Depression Rating Scale (HAM-D) [59]	Gastrointestinal somatic symptoms: “Loss of appetite but eating without staff encouragement. Heavy feelings in abdomen.”	Loss of weight: according to the patient or weekly measurements.		
Montgomery–Asberg Depression Rating Scale (MADRS) [61]	Reduced appetite: representing the feeling of a loss of appetite compared with when well. Rate by loss of desire for food or the need to force oneself to eat.			
Center For Epidemiological Studies—Depression (CES-D) [77]	I did not feel like eating; my appetite was poor			
MENO-D [72]				Have you gained weight (in comparison to pre-menopause weight)?
Patient Health Questionnaire (PHQ-9) [62]	Poor appetite or overeating			
Zung Self-Rating Depressive Scale [63]	I eat as much as I used to			
Patient-Reported Outcomes Measurement Information System (PROMIS) [78]	None			
Hamilton Depression Rating Scale (HAM-7) [79]	None			
The Hospital Anxiety and Depression Scale [80]	None			
Edinburgh Postnatal Depression Scale (EDPS) [69]	None			
Geriatric Depression Scale (GDS) [70]	None			
Calgary Depression Scale For Schizophrenia (CDSS) [71]	None			

## 6. Conclusions and Comments

As MDD and obesity frequently co-occur and have significant negative consequences in individuals and society, it is of great importance to better understand the relationship between them. In this Perspective article we have demonstrated how the current use of rating scales limits our understanding of the relationship between MDD, antidepressants, and obesity. Some of the most employed depressive-symptoms questionnaires lack items that address weight and appetite increments. Considering that most antidepressant trials have overlooked BMI measurements and utilize MADRS and HAM-D, they are limited to appetite- and weight-loss items as we show in Table 1. Scales including appetite and weight increase, as well as thorough BMI and metabolic markers, are needed to establish the relationship between antidepressants, weight, and metabolism. We encourage future research studies to prospectively study—from the beginning, during, and after the interventions—weight and eating behaviors in MDD. We consider that this may improve our understanding of weight gain in MDD as previous systematic reviews have also reported the lack of consistency in these measurements to properly study the relationship between MDD, obesity, and antidepressants [3,57]. Also, it would be valuable for the appropriate appetite and weight items to be added to current and future questionnaires. It is important to note that the search performed in this Perspective article was not systematized and was limited to PubMed and questionnaires in English. Future research may also benefit from a systematic review on the topic.
